# Supplementary material for: A potent KRAS macromolecule degrader specifically targeting tumours with mutant KRAS
Source: Nat Commun. 2020 Jun 26;11:3233. doi: 10.1038/s41467-020-17022-w (PMC7319959; doi:10.1038/s41467-020-17022-w)
Supplement: Supplementary file 1 — Supplementary Information [file 41467_2020_17022_MOESM1_ESM.pdf]

Supplementary Information

A potent KRAS macromolecule degrader specifically targeting tumours with mutant KRAS

Bery, N et al.

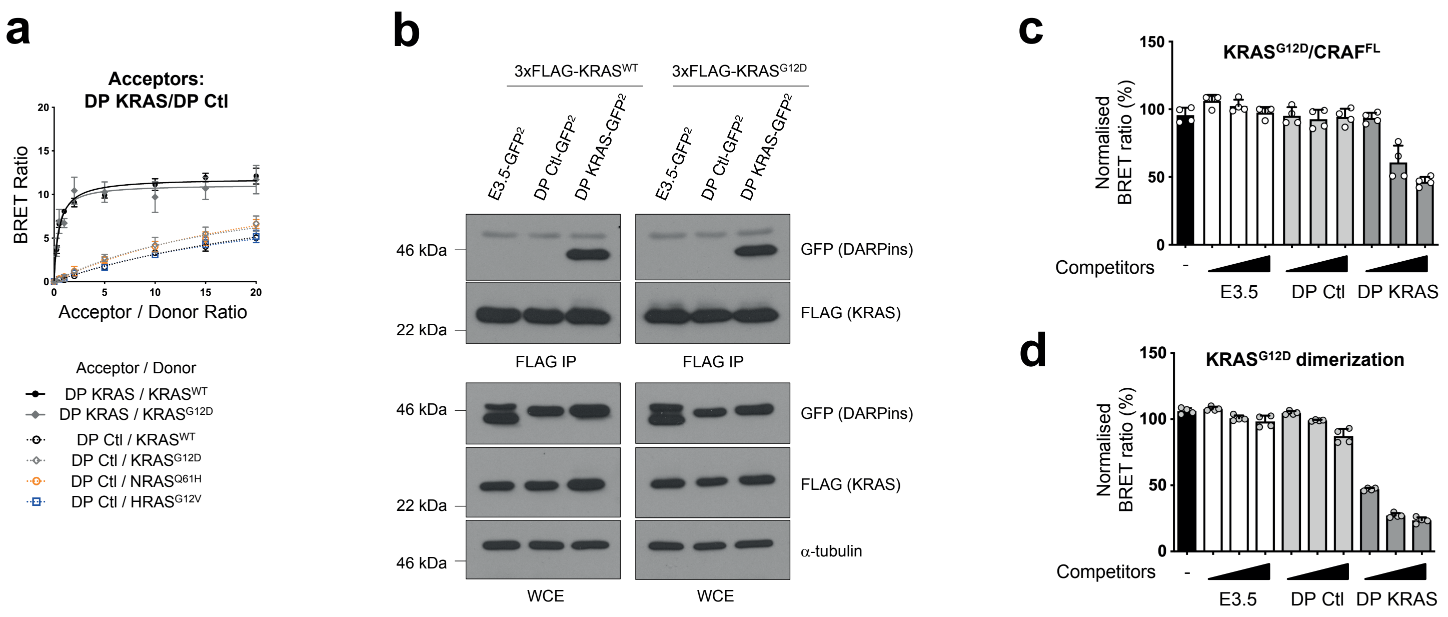

e

iDAb RAS-UBOX DNA and protein sequences

```
        10        20        30        40        50        60        70        80        90       100
ATGCTGTGCTGTATGAGAAGAACCAAACAGGTTGAAAAGAATGATGAGGACCAAAAGATCGTCGACATGGACTACAAAGACGACGATGACAGGCCCATGG
TACGACACGACATACTCTTCTTGGTTTGTCCAACTTTTCTTACTACTCCTGGTTTTCTAGCAGCTGTACCTGATGTTTCTGCTGCTACTGTCCGGGTACC
 M  L  C  C  M  R  R  T  K  Q  V  E  K  N  D  E  D  Q  K  I  V  D  M  D  Y  K  D  D  D  D  R  P  M>

       110       120       130       140       150       160       170       180       190       200
CCGAGGTGCAGCTGTTGGAGTCTGGGGGAGGCTTGGTACAGCCTGGGGGGTCCCTGAGACTCTCCTGTGCAGCCTCTGGATTCACCTTTAGTACCTTTAG
GGCTCCACGTCGACAACCTCAGACCCCCTCCGAACCATGTCGGACCCCCCAGGGACTCTGAGAGGACACGTCGGAGACCTAAGTGGAAATCATGGAAATC
A  E  V  Q  L  L  E  S  G  G  G  L  V  Q  P  G  G  S  L  R  L  S  C  A  A  S  G  F  T  F  S  T  F  S>

       210       220       230       240       250       260       270       280       290       300
CATGAACTGGGTCCGCCAGGCTCCAGGGAAGGGGCTGGAGTGGGTTTCATACATTAGTAGGACGTCGAAGACGATATACTATGCAGACTCTGTGAAGGGC
GTACTTGACCCAGGCGGTCCGAGGTCCCTTCCCCGACCTCACCCAAAGTATGTAATCATCCTGCAGCTTCTGCTATATGATACGTCTGAGACACTTCCCG
  M  N  W  V  R  Q  A  P  G  K  G  L  E  W  V  S  Y  I  S  R  T  S  K  T  I  Y  Y  A  D  S  V  K  G>

       310       320       330       340       350       360       370       380       390       400
CGATTCACCATCTCCAGAGACAATTCCAAGAACACACTGTATCTGCAAATGAACAGCCTGAGAGCCGAGGACACGGCTGTCTATTACTGTGCGAGAGGGA
GCTAAGTGGTAGAGGTCTCTGTTAAGGTTCTTGTGTGACATAGACGTTTACTTGTCGGACTCTCGGCTCCTGTGCCGACAGATAATGACACGCTCTCCCT
 R  F  T  I  S  R  D  N  S  K  N  T  L  Y  L  Q  M  N  S  L  R  A  E  D  T  A  V  Y  Y  C  A  R  G>

       410       420       430       440       450       460       470       480       490       500
GATTCTTTGACTACTGGGGCCAGGGAACCCTGGTCACCGTTAGTTCTCTCGAGGGCGGAGGCGGATCTGCGGCCGCTCGGCTGAACTTCGGGGACGACAT
CTAAGAAACTGATGACCCCGGTCCCTTGGGACCAGTGGCAATCAAGAGAGCTCCCGCCTCCGCCTAGACGCCGGCGAGCCGACTTGAAGCCCCTGCTGTA
R  F  F  D  Y  W  G  Q  G  T  L  V  T  V  S  S  L  E  G  G  G  G  S  A  A  A  R  L  N  F  G  D  D  I>

       510       520       530       540       550       560       570       580       590       600
CCCCAGCGCTCTTCGAATCGCGAAGAAGAAGCGCTGGAACAGCATTGAGGAGCGGCGCATCCACCAGGAGAGCGAGCTGCACTCCTACCTCTCCAGGCTC
GGGGTCGCGAGAAGCTTAGCGCTTCTTCTTCGCGACCTTGTCGTAACTCCTCGCCGCGTAGGTGGTCCTCTCGCTCGACGTGAGGATGGAGAGGTCCGAG
  P  S  A  L  R  I  A  K  K  K  R  W  N  S  I  E  E  R  R  I  H  Q  E  S  E  L  H  S  Y  L  S  R  L>

       610       620       630       640       650       660       670       680       690       700
ATTGCCGCGGAGCGTGAGAGGGAGCTGGAAGAGTGCCAGCGAAACCACGAGGGTGATGAGGACGACAGCCACGTCCGGGCCCAGCAGGCCTGCATTGAGG
TAACGGCGCCTCGCACTCTCCCTCGACCTTCTCACGGTCGCTTTGGTGCTCCCACTACTCCTGCTGTCGGTGCAGGCCCGGGTCGTCCGGACGTAACTCC
 I  A  A  E  R  E  R  E  L  E  E  C  Q  R  N  H  E  G  D  E  D  D  S  H  V  R  A  Q  Q  A  C  I  E>

       710       720       730       740       750       760       770       780       790       800
CCAAGCACGACAAGTACATGGCGGACATGGACGAGCTTTTTTCTCAGGTGGATGAGAAGAGGAAGAAGCGAGACATCCCCGACTACCTGTGTGGCAAGAT
GGTTCGTGCTGTTCATGTACCGCCTGTACCTGCTCGAAAAAAGAGTCCACCTACTCTTCTCCTTCTTCGCTCTGTAGGGGCTGATGGACACACCGTTCTA
A  K  H  D  K  Y  M  A  D  M  D  E  L  F  S  Q  V  D  E  K  R  K  K  R  D  I  P  D  Y  L  C  G  K  I>

       810       820       830       840       850       860       870       880       890       900
CAGCTTTGAGCTGATGCGGGAGCCGTGCATCACGCCCAGTGGCATCACCTACGACCGCAAGGACATCGAGGAGCACCTGCAGCGTGTGGGTCATTTTGAC
GTCGAAACTCGACTACGCCCTCGGCACGTAGTGCGGGTCACCGTAGTGGATGCTGGCGTTCCTGTAGCTCCTCGTGGACGTCGCACACCCAGTAAAACTG
  S  F  E  L  M  R  E  P  C  I  T  P  S  G  I  T  Y  D  R  K  D  I  E  E  H  L  Q  R  V  G  H  F  D>

       910       920       930       940       950       960       970       980       990      1000
CCCGTGACCCGGAGCCCCCTGACCCAGGAACAGCTCATCCCCAACTTGGCTATGAAGGAGGTTATTGACGCATTCATCTCTGAGAATGGCTGGGTGGAGG
GGGCACTGGGCCTCGGGGGACTGGGTCCTTGTCGAGTAGGGGTTGAACCGATACTTCCTCCAATAACTGCGTAAGTAGAGACTCTTACCGACCCACCTCC
 P  V  T  R  S  P  L  T  Q  E  Q  L  I  P  N  L  A  M  K  E  V  I  D  A  F  I  S  E  N  G  W  V  E>

ACTACTGA
TGATGACT
D  Y  *>
```

Supplementary Figure 1: Characterisation of DARPin control and the degraders. (a) BRET donor saturation assay between DARPin KRAS (DP KRAS) or DARPin Control (DP Ctl) as acceptor and $KRAS^{WT}$, $KRAS^{G12D}$, $NRAS^{Q61H}$ or $HRAS^{G12V}$ as donors. (b) Co-immunoprecipitation of 3xFLAG-$KRAS^{WT}$ and 3xFLAG-$KRAS^{G12D}$ with the DARPin-GFP^2 fusions in 10% foetal bovine serum. IP: Immunoprecipitation, WCE: whole-cell extract. DARPin E3.5 is a non-relevant DARPin. (c) BRET competition by the indicated DARPins of interaction between $KRAS^{G12D}$ and $CRAF^{FL}$ or (d) $KRAS^{G12D}$ dimerization. (-) means no competitor was used. Each experiment was performed twice (a, b) or four times (c, d). Error bars are mean ± SD of biological repeats (a, c, d). (e) DNA and protein sequences of iDAb RAS-UBOX (pan-RAS degrader). Orange amino acids: iDAb RAS, grey amino acids: glycine/serine linker, blue amino acids: UBOX. **b-d** Source data are provided as a Source Data file.

iDAb Ctl-UBOX DNA and protein sequences

```
         10         20         30         40         50         60         70         80         90        100
ATGCTGTGCTGTATGAGAAGAACCAAACAGGTTGAAAAGAATGATGAGGACCAAAAGATCGTCGACATGGACTACAAAGACGACGATGACAGGCCCATGG
TACGACACGACATACTCTTCTTGGTTTGTCCAACTTTTCTTACTACTCCTGGTTTTCTAGCAGCTGTACCTGATGTTTCTGCTGCTACTGTCCGGGTACC
 M  L  C  C  M  R  R  T  K  Q  V  E  K  N  D  E  D  Q  K  I  V  D  M  D  Y  K  D  D  D  D  R  P  M>

        110        120        130        140        150        160        170        180        190        200
CCGAGGTGCAGCTGTTGGAGTCTGGGGGAGGCTTGGTACAGCCTGGGGGGTCCCTGAGACTCTCCTGTGCAGCCTCTGGATTCAGCTTCAGTCATAGTCC
GGCTCCACGTCGACAACCTCAGACCCCCTCCGAACCATGTCGGACCCCCCAGGGACTCTGAGAGGACACGTCGGAGACCTAAGTCGAAGTCAGTATCAGG
A  E  V  Q  L  L  E  S  G  G  G  L  V  Q  P  G  G  S  L  R  L  S  C  A  A  S  G  F  S  F  S  H  S  P>

        210        220        230        240        250        260        270        280        290        300
TATGAATTGGGTCCGCCAGGCTCCAGGGAAGGGGCTGGAGTGGGTTTCATACATTAGTTATAATTCTTCGAGTATATACTATGCAGACTCTGTGAAGGGC
ATACTTAACCCAGGCGGTCCGAGGTCCCTTCCCCGACCTCACCCAAAGTATGTAATCAATATTAAGAAGCTCATATATGATACGTCTGAGACACTTCCCG
  M  N  W  V  R  Q  A  P  G  K  G  L  E  W  V  S  Y  I  S  Y  N  S  S  S  I  Y  Y  A  D  S  V  K  G>

        310        320        330        340        350        360        370        380        390        400
CGATTCACCATCTCCAGAGACAATTCCAAGAACACACTGTATCTGCAAATGAACAGCCTGAGAGCCGAGGACACGGCTGTCTATTACTGTGCGAGAGGGT
GCTAAGTGGTAGAGGTCTCTGTTAAGGTTCTTGTGTGACATAGACGTTTACTTGTCGGACTCTCGGCTCCTGTGCCGACAGATAATGACACGCTCTCCCA
 R  F  T  I  S  R  D  N  S  K  N  T  L  Y  L  Q  M  N  S  L  R  A  E  D  T  A  V  Y  Y  C  A  R  G>

        410        420        430        440        450        460        470        480        490        500
TGACGGAGTCTCTTGAGTTGACGGCGGATTGGTTTGATTACTGGGGCCAGGGAACCCTGGTCACCGTTAGTTCTCTCGAGGGCGGAGGCGGATCTGCGGC
ACTGCCTCAGAGAACTCAACTGCCGCCTAACCAAACTAATGACCCCGGTCCCTTGGGACCAGTGGCAATCAAGAGAGCTCCCGCCTCCGCCTAGACGCCG
L  T  E  S  L  E  L  T  A  D  W  F  D  Y  W  G  Q  G  T  L  V  T  V  S  S  L  E  G  G  G  G  S  A  A>

        510        520        530        540        550        560        570        580        590        600
CGCTCGGCTGAACTTCGGGGACGACATCCCCAGCGCTCTTCGAATCGCGAAGAAGAAGCGCTGGAACAGCATTGAGGAGCGGCGCATCCACCAGGAGAGC
GCGAGCCGACTTGAAGCCCCTGCTGTAGGGGTCGCGAGAAGCTTAGCGCTTCTTCTTCGCGACCTTGTCGTAACTCCTCGCCGCGTAGGTGGTCCTCTCG
  A  R  L  N  F  G  D  D  I  P  S  A  L  R  I  A  K  K  K  R  W  N  S  I  E  E  R  R  I  H  Q  E  S>

        610        620        630        640        650        660        670        680        690        700
GAGCTGCACTCCTACCTCTCCAGGCTCATTGCCGCGGAGCGTGAGAGGGAGCTGGAAGAGTGCCAGCGAAACCACGAGGGTGATGAGGACGACAGCCACG
CTCGACGTGAGGATGGAGAGGTCCGAGTAACGGCGCCTCGCACTCTCCCTCGACCTTCTCACGGTCGCTTTGGTGCTCCCACTACTCCTGCTGTCGGTGC
 E  L  H  S  Y  L  S  R  L  I  A  A  E  R  E  R  E  L  E  E  C  Q  R  N  H  E  G  D  E  D  D  S  H>

        710        720        730        740        750        760        770        780        790        800
TCCGGGCCCAGCAGGCCTGCATTGAGGCCAAGCACGACAAGTACATGGCGGACATGGACGAGCTTTTTTCTCAGGTGGATGAGAAGAGGAAGAAGCGAGA
AGGCCCGGGTCGTCCGGACGTAACTCCGGTTCGTGCTGTTCATGTACCGCCTGTACCTGCTCGAAAAAAGAGTCCACCTACTCTTCTCCTTCTTCGCTCT
V  R  A  Q  Q  A  C  I  E  A  K  H  D  K  Y  M  A  D  M  D  E  L  F  S  Q  V  D  E  K  R  K  K  R  D>

        810        820        830        840        850        860        870        880        890        900
CATCCCCGACTACCTGTGTGGCAAGATCAGCTTTGAGCTGATGCGGGAGCCGTGCATCACGCCCAGTGGCATCACCTACGACCGCAAGGACATCGAGGAG
GTAGGGGCTGATGGACACACCGTTCTAGTCGAAACTCGACTACGCCCTCGGCACGTAGTGCGGGTCACCGTAGTGGATGCTGGCGTTCCTGTAGCTCCTC
  I  P  D  Y  L  C  G  K  I  S  F  E  L  M  R  E  P  C  I  T  P  S  G  I  T  Y  D  R  K  D  I  E  E>

        910        920        930        940        950        960        970        980        990       1000
CACCTGCAGCGTGTGGGTCATTTTGACCCCGTGACCCGGAGCCCCCTGACCCAGGAACAGCTCATCCCCAACTTGGCTATGAAGGAGGTTATTGACGCAT
GTGGACGTCGCACACCCAGTAAAACTGGGGCACTGGGCCTCGGGGGACTGGGTCCTTGTCGAGTAGGGGTTGAACCGATACTTCCTCCAATAACTGCGTA
 H  L  Q  R  V  G  H  F  D  P  V  T  R  S  P  L  T  Q  E  Q  L  I  P  N  L  A  M  K  E  V  I  D  A>

       1010       1020       1030
TCATCTCTGAGAATGGCTGGGTGGAGGACTACTGA
AGTAGAGACTCTTACCGACCCACCTCCTGATGACT
F  I  S  E  N  G  W  V  E  D  Y  *>
```

Supplementary Figure 2: DNA and protein sequences of iDAb Ctl-UBOX. Orange amino acids: iDAb Ctl, grey amino acids: glycine/serine linker, blue amino acids: UBOX.

VHL-DP KRAS DNA and protein sequences

```
        10        20        30        40        50        60        70        80        90       100
ATGCCCCGGAGGGCGGAGAACTGGGACGAGGCCGAGGTAGGCGCGGAGGAGGCAGGCGTCGAAGAGTACGGCCCTGAAGAAGACGGCGGGGAGGAGTCGG
TACGGGGCCTCCCGCCTCTTGACCCTGCTCCGGCTCCATCCGCGCCTCCTCCGTCCGCAGCTTCTCATGCCGGGACTTCTTCTGCCGCCCCTCCTCAGCC
 M  P  R  R  A  E  N  W  D  E  A  E  V  G  A  E  E  A  G  V  E  E  Y  G  P  E  E  D  G  G  E  E  S>

       110       120       130       140       150       160       170       180       190       200
GCGCCGAGGAGTCCGGCCCGGAAGAGTCCGGCCCGGAGGAACTGGGCGCCGAGGAGGAGATGGAGGCCGGGCGGCCGCGGCCCGTGCTGCGCTCGGTGAA
CGCGGCTCCTCAGGCCGGGCCTTCTCAGGCCGGGCCTCCTTGACCCGCGGCTCCTCCTCTACCTCCGGCCCGCCGGCGCCGGGCACGACGCGAGCCACTT
G  A  E  E  S  G  P  E  E  S  G  P  E  E  L  G  A  E  E  E  M  E  A  G  R  P  R  P  V  L  R  S  V  N>

       210       220       230       240       250       260       270       280       290       300
CTCGCGCGAGCCCTCCCAGGTCATCTTCTGCAATCGCAGTCCGCGCGTCGTGCTGCCAGTATGGCTCAACTTCGACGGCGAGCCGCAGCCCTACCCAACG
GAGCGCGCTCGGGAGGGTCCAGTAGAAGACGTTAGCGTCAGGCGCGCAGCACGACGGTCATACCGAGTTGAAGCTGCCGCTCGGCGTCGGGATGGGTTGC
  S  R  E  P  S  Q  V  I  F  C  N  R  S  P  R  V  V  L  P  V  W  L  N  F  D  G  E  P  Q  P  Y  P  T>

       310       320       330       340       350       360       370       380       390       400
CTGCCGCCTGGCACGGGCCGCCGCATCCACAGCTACCGAGGTCACCTTTGGCTCTTCAGAGATGCAGGGACACACGATGGGCTTCTGGTTAACCAAACTG
GACGGCGGACCGTGCCCGGCGGCGTAGGTGTCGATGGCTCCAGTGGAAACCGAGAAGTCTCTACGTCCCTGTGTGCTACCCGAAGACCAATTGGTTTGAC
 L  P  P  G  T  G  R  R  I  H  S  Y  R  G  H  L  W  L  F  R  D  A  G  T  H  D  G  L  L  V  N  Q  T>

       410       420       430       440       450       460       470       480       490       500
AATTATTTGTGCCATCTCTCAATGTTGACGGACAGCCTATTTTTGCCAATATCACACTGCCAGTGTATACTCTGAAAGAGCGATGCCTCCAGGTTGTCCG
TTAATAAACACGGTAGAGAGTTACAACTGCCTGTCGGATAAAAACGGTTATAGTGTGACGGTCACATATGAGACTTTCTCGCTACGGAGGTCCAACAGGC
E  L  F  V  P  S  L  N  V  D  G  Q  P  I  F  A  N  I  T  L  P  V  Y  T  L  K  E  R  C  L  Q  V  V  R>

       510       520       530       540       550       560       570       580       590       600
GAGCCTAGTCAAGCCTGAGAATTACAGGAGACTGGACATCGTCAGGTCGCTCTACGAAGATCTGGAAGACCACCCAAATGTGCAGAAAGACCTGGAGCGG
CTCGGATCAGTTCGGACTCTTAATGTCCTCTGACCTGTAGCAGTCCAGCGAGATGCTTCTAGACCTTCTGGTGGGTTTACACGTCTTTCTGGACCTCGCC
  S  L  V  K  P  E  N  Y  R  R  L  D  I  V  R  S  L  Y  E  D  L  E  D  H  P  N  V  Q  K  D  L  E  R>

       610       620       630       640       650       660       670       680       690       700
CTGACACAGGAGCGCATTGCACATCAACGGATGGGAGATCTCGAGGGCGGAGGCGGATCTGCGGCCGCAATGGATCTGGGAAAAAAACTGCTGGAAGCCG
GACTGTGTCCTCGCGTAACGTGTAGTTGCCTACCCTCTAGAGCTCCCGCCTCCGCCTAGACGCCGGCGTTACCTAGACCCTTTTTTTGACGACCTTCGGC
 L  T  Q  E  R  I  A  H  Q  R  M  G  D  L  E  G  G  G  G  S  A  A  A  M  D  L  G  K  K  L  L  E  A>

       710       720       730       740       750       760       770       780       790       800
CGCGTGCCGGGCAGGACGATGAGGTCCGTATTCTTATGGCGAATGGTGCAGATGTTAACGCGAGCGATCGTTGGGGTTGGACGCCGCTGCACCTGGCAGC
GCGCACGGCCCGTCCTGCTACTCCAGGCATAAGAATACCGCTTACCACGTCTACAATTGCGCTCGCTAGCAACCCCAACCTGCGGCGACGTGGACCGTCG
A  R  A  G  Q  D  D  E  V  R  I  L  M  A  N  G  A  D  V  N  A  S  D  R  W  G  W  T  P  L  H  L  A  A>

       810       820       830       840       850       860       870       880       890       900
GTGGTGGGGTCACCTCGAAATTGTGGAAGTGCTGTTGAAGCGCGGTGCAGATGTTAGCGCGGCAGATCTGCACGGTCAATCGCCGCTGCATCTGGCAGCG
CACCACCCCAGTGGAGCTTTAACACCTTCACGACAACTTCGCGCCACGTCTACAATCGCGCCGTCTAGACGTGCCAGTTAGCGGCGACGTAGACCGTCGC
  W  W  G  H  L  E  I  V  E  V  L  L  K  R  G  A  D  V  S  A  A  D  L  H  G  Q  S  P  L  H  L  A  A>

       910       920       930       940       950       960       970       980       990      1000
ATGGTCGGCCACCTCGAAATTGTGGAAGTGCTGTTGAAGTACGGTGCAGATGTTAACGCGAAAGATACGATGGGTGCAACGCCGCTGCACCTGGCAGCGC
TACCAGCCGGTGGAGCTTTAACACCTTCACGACAACTTCATGCCACGTCTACAATTGCGCTTTCTATGCTACCCACGTTGCGGCGACGTGGACCGTCGCG
 M  V  G  H  L  E  I  V  E  V  L  L  K  Y  G  A  D  V  N  A  K  D  T  M  G  A  T  P  L  H  L  A  A>

      1010      1020      1030      1040      1050      1060      1070      1080      1090      1100
GAAGCGGTCACCTCGAAATTGTGGAAGAGCTGTTGAAGAACGGTGCAGATATGAATGCTCAGGATAAGTTTGGCAAAACCACGTTTGATATCTCCACTGA
CTTCGCCAGTGGAGCTTTAACACCTTCTCGACAACTTCTTGCCACGTCTATACTTACGAGTCCTATTCAAACCGTTTTGGTGCAAACTATAGAGGTGACT
R  S  G  H  L  E  I  V  E  E  L  L  K  N  G  A  D  M  N  A  Q  D  K  F  G  K  T  T  F  D  I  S  T  D>

      1110      1120      1130      1140      1150      1160      1170      1180
TAATGGCAACGAAGATTTAGCGGAAATCCTGCAGAAACTGGTCGACGGCGGGTCTGACTACAAAGACGACGATGACAAGTAA
ATTACCGTTGCTTCTAAATCGCCTTTAGGACGTCTTTGACCAGCTGCCGCCCAGACTGATGTTTCTGCTGCTACTGTTCATT
  N  G  N  E  D  L  A  E  I  L  Q  K  L  V  D  G  G  S  D  Y  K  D  D  D  D  K  *>
```

Supplementary Figure 3: DNA and protein sequences of VHL-DP KRAS (KRAS degrader). Orange amino acids: DP KRAS, grey amino acids: glycine/serine linker, blue amino acids: VHL.

VHL-DP Ctl DNA and protein sequences

```
         10         20         30         40         50         60         70         80         90        100
ATGCCCCGGAGGGCGGAGAACTGGGACGAGGCCGAGGTAGGCGCGGAGGAGGCAGGCGTCGAAGAGTACGGCCCTGAAGAAGACGGCGGGGAGGAGTCGG
TACGGGGCCTCCCGCCTCTTGACCCTGCTCCGGCTCCATCCGCGCCTCCTCCGTCCGCAGCTTCTCATGCCGGGACTTCTTCTGCCGCCCCTCCTCAGCC
 M  P  R  R  A  E  N  W  D  E  A  E  V  G  A  E  E  A  G  V  E  E  Y  G  P  E  E  D  G  G  E  E  S>

        110        120        130        140        150        160        170        180        190        200
GCGCCGAGGAGTCCGGCCCGGAAGAGTCCGGCCCGGAGGAACTGGGCGCCGAGGAGGAGATGGAGGCCGGGCGGCCGCGGCCCGTGCTGCGCTCGGTGAA
CGCGGCTCCTCAGGCCGGGCCTTCTCAGGCCGGGCCTCCTTGACCCGCGGCTCCTCCTCTACCTCCGGCCCGCCGGCGCCGGGCACGACGCGAGCCACTT
G  A  E  E  S  G  P  E  E  S  G  P  E  E  L  G  A  E  E  E  M  E  A  G  R  P  R  P  V  L  R  S  V  N>

        210        220        230        240        250        260        270        280        290        300
CTCGCGCGAGCCCTCCCAGGTCATCTTCTGCAATCGCAGTCCGCGCGTCGTGCTGCCCGTATGGCTCAACTTCGACGGCGAGCCGCAGCCCTACCCAACG
GAGCGCGCTCGGGAGGGTCCAGTAGAAGACGTTAGCGTCAGGCGCGCAGCACGACGGGCATACCGAGTTGAAGCTGCCGCTCGGCGTCGGGATGGGTTGC
  S  R  E  P  S  Q  V  I  F  C  N  R  S  P  R  V  V  L  P  V  W  L  N  F  D  G  E  P  Q  P  Y  P  T>

        310        320        330        340        350        360        370        380        390        400
CTGCCGCCTGGCACGGGCCGCCGCATCCACAGCTACCGAGGTCACCTTTGGCTCTTCAGAGATGCAGGGACACACGATGGGCTTCTGGTTAACCAAACTG
GACGGCGGACCGTGCCCGGCGGCGTAGGTGTCGATGGCTCCAGTGGAAACCGAGAAGTCTCTACGTCCCTGTGTGCTACCCGAAGACCAATTGGTTTGAC
 L  P  P  G  T  G  R  R  I  H  S  Y  R  G  H  L  W  L  F  R  D  A  G  T  H  D  G  L  L  V  N  Q  T>

        410        420        430        440        450        460        470        480        490        500
AATTATTTGTGCCATCTCTCAATGTTGACGGACAGCCTATTTTTGCCAATATCACACTGCCAGTGTATACTCTGAAAGAGCGATGCCTCCAGGTTGTCCG
TTAATAAACACGGTAGAGAGTTACAACTGCCTGTCGGATAAAAACGGTTATAGTGTGACGGTCACATATGAGACTTTCTCGCTACGGAGGTCCAACAGGC
E  L  F  V  P  S  L  N  V  D  G  Q  P  I  F  A  N  I  T  L  P  V  Y  T  L  K  E  R  C  L  Q  V  V  R>

        510        520        530        540        550        560        570        580        590        600
GAGCCTAGTCAAGCCTGAGAATTACAGGAGACTGGACATCGTCAGGTCGCTCTACGAAGATCTGGAAGACCACCCAAATGTGCAGAAAGACCTGGAGCGG
CTCGGATCAGTTCGGACTCTTAATGTCCTCTGACCTGTAGCAGTCCAGCGAGATGCTTCTAGACCTTCTGGTGGGTTTACACGTCTTTCTGGACCTCGCC
  S  L  V  K  P  E  N  Y  R  R  L  D  I  V  R  S  L  Y  E  D  L  E  D  H  P  N  V  Q  K  D  L  E  R>

        610        620        630        640        650        660        670        680        690        700
CTGACACAGGAGCGCATTGCACATCAACGGATGGGAGATCTCGAGGGCGGAGGCGGATCTGCGGCCGCAATGGATCTGGGAAAAAAACTGCTGGAAGCCG
GACTGTGTCCTCGCGTAACGTGTAGTTGCCTACCCTCTAGAGCTCCCGCCTCCGCCTAGACGCCGGCGTTACCTAGACCCTTTTTTTGACGACCTTCGGC
 L  T  Q  E  R  I  A  H  Q  R  M  G  D  L  E  G  G  G  G  S  A  A  A  M  D  L  G  K  K  L  L  E  A>

        710        720        730        740        750        760        770        780        790        800
CGCGTGCCGGGCAGGACGATGAGGTCCGTATTCTTATGGCGAATGGTGCAGATGTTAACGCGAGCGATCGCGGGGGTGGCACGCCGCTGCACCTGGCAGC
GCGCACGGCCCGTCCTGCTACTCCAGGCATAAGAATACCGCTTACCACGTCTACAATTGCGCTCGCTAGCGCCCCCACCGTGCGGCGACGTGGACCGTCG
A  R  A  G  Q  D  D  E  V  R  I  L  M  A  N  G  A  D  V  N  A  S  D  R  G  G  G  T  P  L  H  L  A  A>

        810        820        830        840        850        860        870        880        890        900
GGCAGGGGGTCACCTCGAAATTGTGGAAGTGCTGTTGAAGCGCGGTGCAGATGTTAGCGCGGCAGATCTGCACGGTCAATCGCCGCTGCATCTGGCAGCG
CCGTCCCCCAGTGGAGCTTTAACACCTTCACGACAACTTCGCGCCACGTCTACAATCGCGCCGTCTAGACGTGCCAGTTAGCGGCGACGTAGACCGTCGC
  A  G  G  H  L  E  I  V  E  V  L  L  K  R  G  A  D  V  S  A  A  D  L  H  G  Q  S  P  L  H  L  A  A>

        910        920        930        940        950        960        970        980        990       1000
ATGGTCGGCCACCTCGAAATTGTGGAAGTGCTGTTGAAGTACGGTGCAGATGTTAACGCGAAAGATACGATGGGTGCAACGCCGCTGCACCTGGCAGCGC
TACCAGCCGGTGGAGCTTTAACACCTTCACGACAACTTCATGCCACGTCTACAATTGCGCTTTCTATGCTACCCACGTTGCGGCGACGTGGACCGTCGCG
 M  V  G  H  L  E  I  V  E  V  L  L  K  Y  G  A  D  V  N  A  K  D  T  M  G  A  T  P  L  H  L  A  A>

       1010       1020       1030       1040       1050       1060       1070       1080       1090       1100
GAAGCGGTCACCTCGAAATTGTGGAAGAGCTGTTGAAGAACGGTGCAGATATGAATGCTCAGGATAAGTTTGGCAAAACCACGTTTGATATCTCCACTGA
CTTCGCCAGTGGAGCTTTAACACCTTCTCGACAACTTCTTGCCACGTCTATACTTACGAGTCCTATTCAAACCGTTTTGGTGCAAACTATAGAGGTGACT
R  S  G  H  L  E  I  V  E  E  L  L  K  N  G  A  D  M  N  A  Q  D  K  F  G  K  T  T  F  D  I  S  T  D>

       1110       1120       1130       1140       1150       1160       1170       1180
TAATGGCAACGAAGATTTAGCGGAAATCCTGCAGAAACTGGTCGACGGCGGGTCTGACTACAAAGACGACGATGACAAGTAA
ATTACCGTTGCTTCTAAATCGCCTTTAGGACGTCTTTGACCAGCTGCCGCCCAGACTGATGTTTCTGCTGCTACTGTTCATT
  N  G  N  E  D  L  A  E  I  L  Q  K  L  V  D  G  G  S  D  Y  K  D  D  D  D  K  *>
```

Supplementary Figure 4: DNA and protein sequences of VHL-DP Ctl. Orange amino acids: DP Ctl, grey amino acids: glycine/serine linker, blue amino acids: VHL.

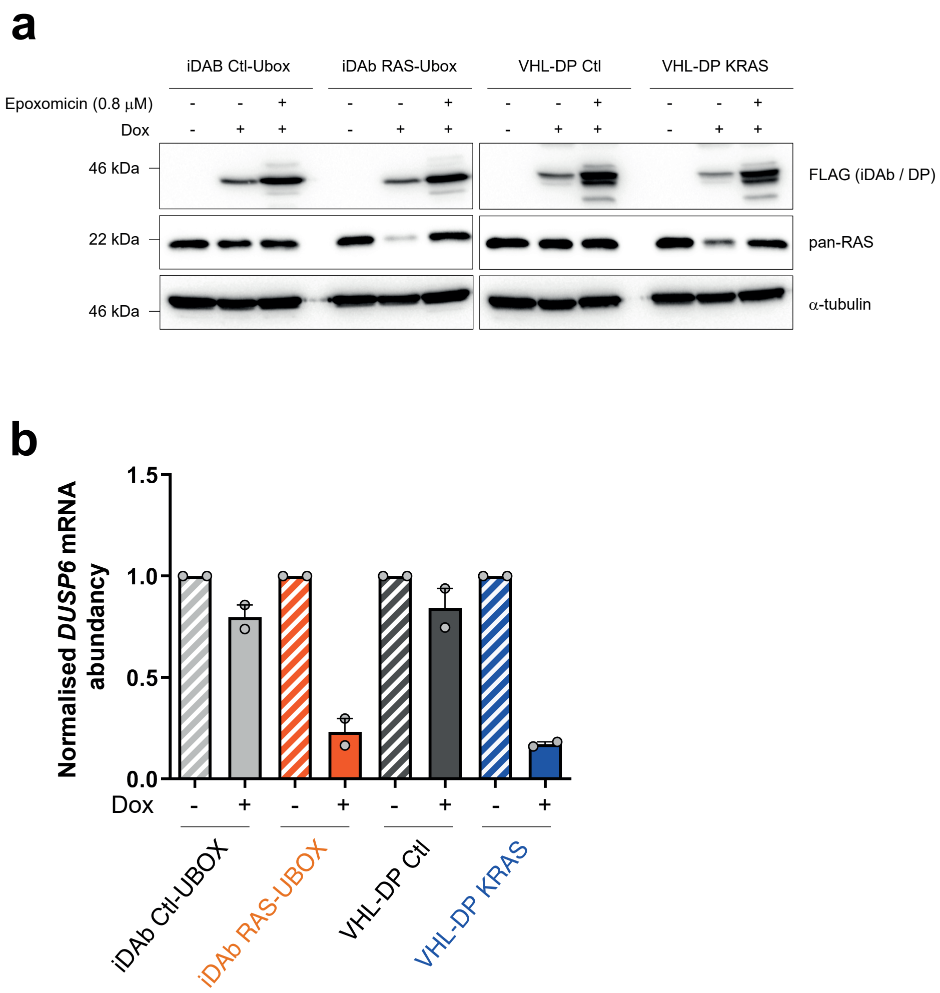

Supplementary Figure 5: RAS degraders induce their endogenous target degradation through the proteasome machinery. (a) H358 cells expressing iDAb Ctl-UBOX, iDAb RAS-UBOX, VHL-DP Ctl or VHL-DP KRAS were either untreated (-), treated with dox only (0.5 µg.mL^{-1}) or treated with dox and epoxomicin (0.8 µM) for 18 hours. RAS protein level was determined by Western blot using a pan-RAS antibody. a-tubulin is the loading control. (b) Quantitative real-time PCR was performed after 24 hours of treatment with 0.2 µg.mL^{-1} of dox (+) or untreated (-) in H358 stable cell lines. No dox conditions were standardised to a value of 1.0 and DUSP6 mRNA abundancy is represented as a fold-change relative to that value. Data are from two independent biological repeats and are normalised to GAPDH. Error bars denote SEM. **a-b** Source data are provided as a Source Data file.

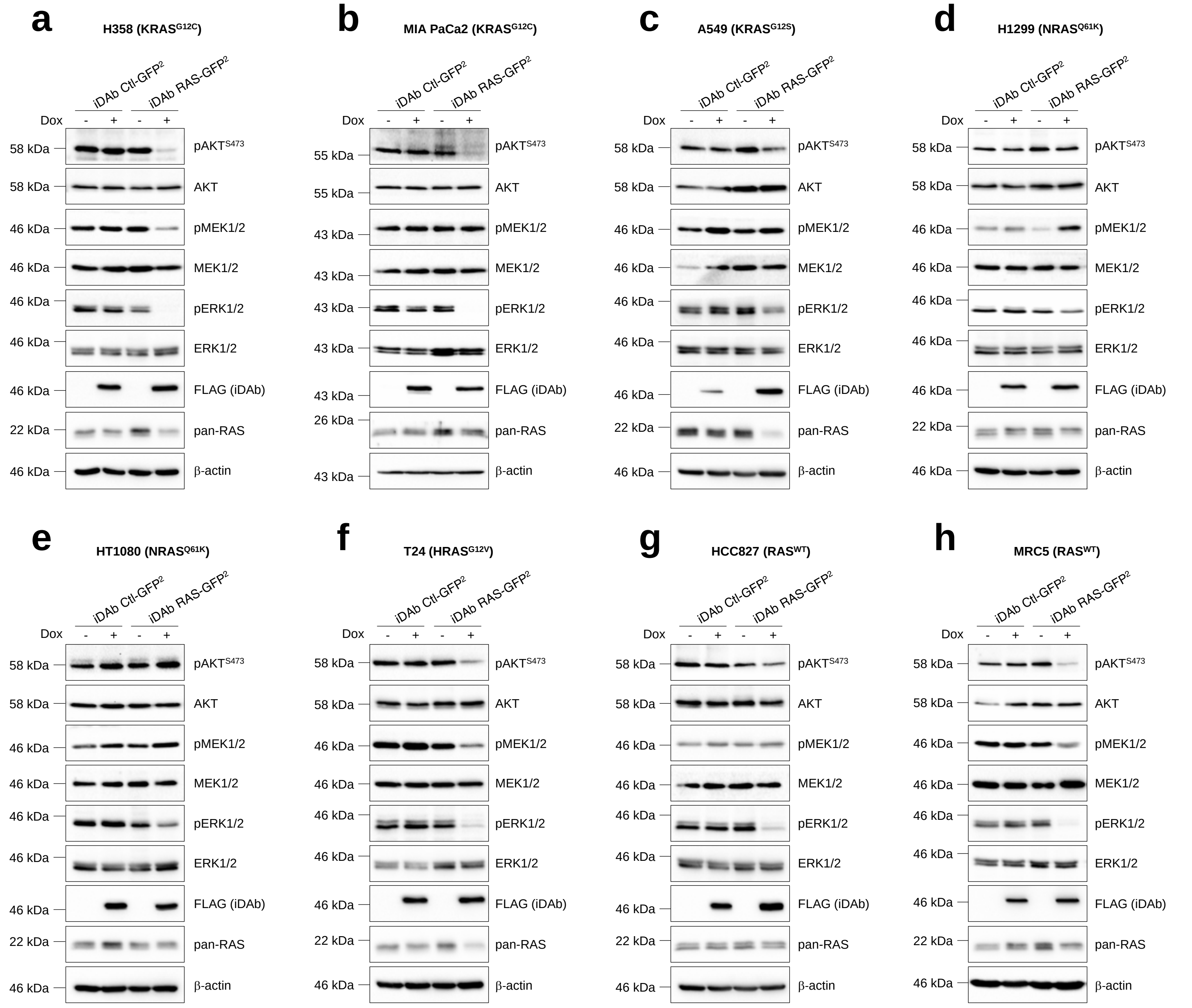

Supplementary Figure 6: Effect of the parental iDAb macrodrug on RAS signalling pathways of various cell lines. Effect of iDAb RAS parental single domain (non-degrader) on RAS signalling pathways of various cell lines: (a) H358 (KRAS^{G12C}), (b) MIA PaCa-2 (KRAS^{G12C}), (c) A549 (KRAS^{G12S}), (d) H1299 (NRAS^{Q61K}), (e) HT1080 (NRAS^{Q61K}),(f) T24 (HRAS^{G12V}), (g) HCC827 (RAS^{WT}) and (h) MRC5 (RAS^{WT}). All the cells stably express dox-inducible iDAb RAS-GFP^2 and its negative control iDAb Ctl-GFP^2. FLAG antibody is used to show iDAb expression when induced with 0.2 μg.mL^{-1} of doxycycline for 72 hours (+) or not induced (-). β-actin is the loading control. Each experiment in (a-h) was performed at least three times. **a-h** Source data are provided as a Source Data file.

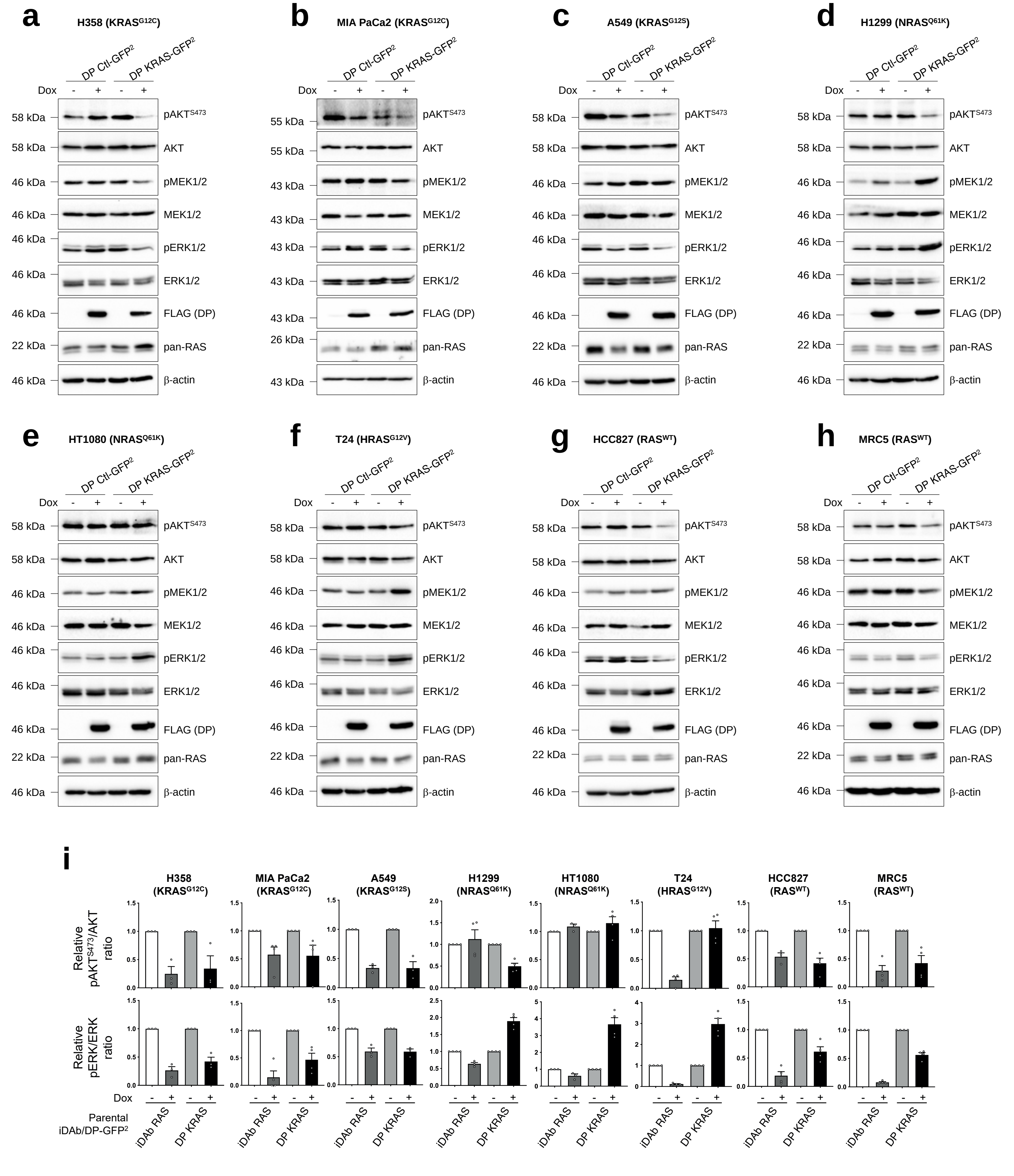

Supplementary Figure 7: Effect of the parental DARPin macrodrug on RAS signalling pathways of various cell lines. The effect of DP KRAS parental single domain (non-degrader) on RAS signalling pathways of various cell lines: (a) H358 ($KRAS^{G12C}$), (b) MIA PaCa-2 ($KRAS^{G12C}$), (c) A549 ($KRAS^{G12S}$), (d) H1299 ($NRAS^{Q61K}$), (e) HT1080 ($NRAS^{Q61K}$), (f) T24 ($HRAS^{G12V}$), (g) HCC827 (RAS^{WT}) and (h) MRC5 (RAS^{WT}). All the cells stably express dox-inducible DP KRAS-GFP^2 and its negative control DP Ctl-GFP^2. FLAG antibody is used to show DARPins (DP) expression when induced with 0.2 $\mu g.mL^{-1}$ of doxycycline for 72 hours (+) or not induced (-). β-actin is the loading control. (i) Comparative quantifications of $pAKT^{S473}$/AKT and pERK/ERK signals affected by iDAb RAS and DP KRAS fused to GFP^2 from Supplementary Figures 6 & 7. The signals were normalised to the no dox (-) condition. Each experiment in (a-h) was performed at least three times. Error bars in (i) are mean ± SEM from at least three biological repeats (n=3 for H358, A549, HT1080 (iDAb RAS condition) and HCC827 for pAKT quantification (iDAb RAS condition), all other cell lines n=4). **a-i** Source data are provided as a Source Data file.

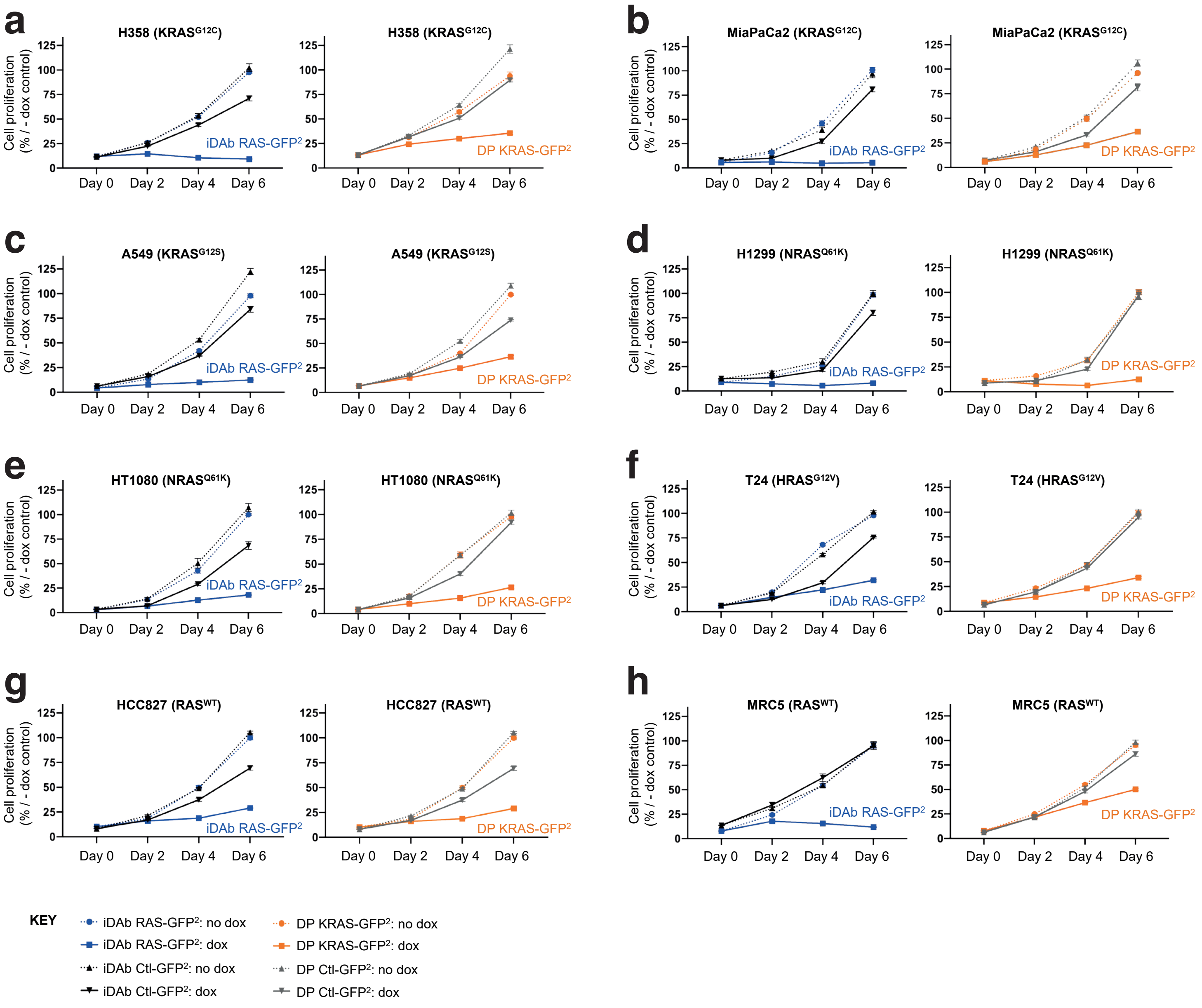

Supplementary Figure 8: Effect of the parental iDAb and DARPin macrodrugs on 2D-adherent proliferation assays of various cell lines. Cells were grown as adherent cultures and macrodrugs induced by doxycycline treatment for assessment of the effect of iDAbs and DPs fused to GFP^2 on proliferation of the mutant KRAS cell lines: (a) H358, (b) MIA PaCa2 and (c) A549. (d-e) Effect of the single domains on 2D-adherent proliferation of NRAS mutant cell lines: (d) H1299 and (e) HT1080. (f) Effect of iDAb-GFP^2 and DP-GFP^2 fusions on 2D-adherent proliferation of mutant HRAS T24 cell lines. (g-h) Effect of the parental single domains on 2D-adherent proliferation of the RAS^{WT} cell lines: (g) HCC827 and (h) MRC5. All proliferation assays were normalised to the no dox condition for each cell line. The plain lines represent the dox-treated cells while the dotted lines show the no dox conditions. Each experiment in (a-h) was performed at least three times. Error bars in (a-h) are mean ± SD from at least three biological repeats (n=3 in b and n=4 in a, c-h). **a-h** Source data are provided as a Source Data file.

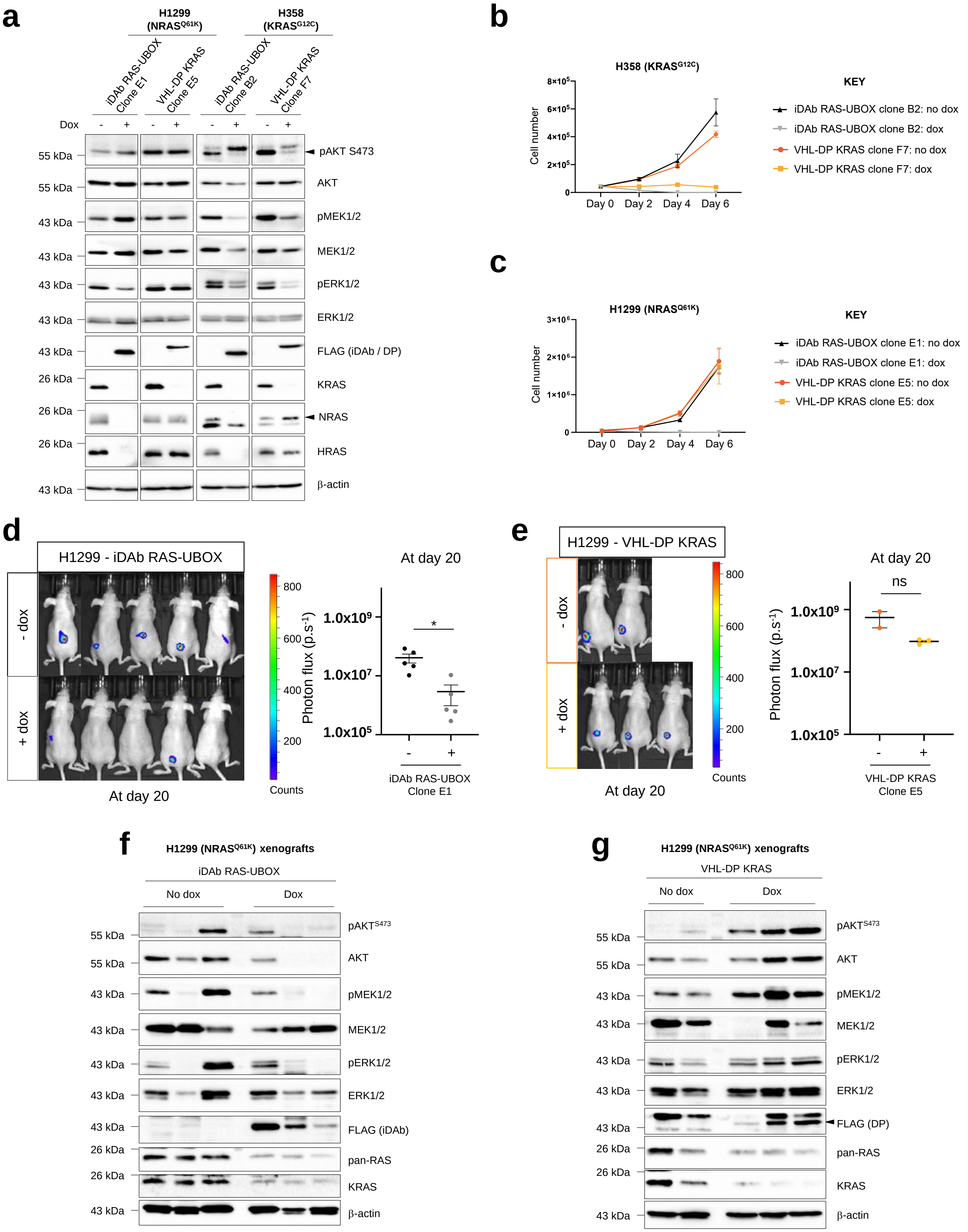

a
H1299 (NRAS^{Q61K})
H358 (KRAS^{G12C})
iDAb RAS-UBOX Clone E1
VHL-DP KRAS Clone E5
iDAb RAS-UBOX Clone B2
VHL-DP KRAS Clone F7
Dox - + - + - + - +
55 kDa pAKT S473
55 kDa AKT
43 kDa pMEK1/2
43 kDa MEK1/2
43 kDa pERK1/2
43 kDa ERK1/2
43 kDa FLAG (iDAb / DP)
26 kDa KRAS
26 kDa NRAS
26 kDa HRAS
43 kDa β-actin
b
H358 (KRAS^{G12C})
Cell number
Day 0 Day 2 Day 4 Day 6
KEY
iDAb RAS-UBOX clone B2: no dox
iDAb RAS-UBOX clone B2: dox
VHL-DP KRAS clone F7: no dox
VHL-DP KRAS clone F7: dox
c
H1299 (NRAS^{Q61K})
Cell number
Day 0 Day 2 Day 4 Day 6
KEY
iDAb RAS-UBOX clone E1: no dox
iDAb RAS-UBOX clone E1: dox
VHL-DP KRAS clone E5: no dox
VHL-DP KRAS clone E5: dox
d
H1299 - iDAb RAS-UBOX
- dox
+ dox
At day 20
800 600 400 200
Counts
At day 20
Photon flux (p.s^{-1})
1.0x10^9
1.0x10^7
1.0x10^5
*
- +
iDAb RAS-UBOX Clone E1
e
H1299 - VHL-DP KRAS
- dox
+ dox
At day 20
800 600 400 200
Counts
At day 20
ns
Photon flux (p.s^{-1})
1.0x10^9
1.0x10^7
1.0x10^5
- +
VHL-DP KRAS Clone E5
f
H1299 (NRAS^{Q61K}) xenografts
iDAb RAS-UBOX
No dox Dox
55 kDa pAKT^{S473}
55 kDa AKT
43 kDa pMEK1/2
43 kDa MEK1/2
43 kDa pERK1/2
43 kDa ERK1/2
43 kDa FLAG (iDAb)
26 kDa pan-RAS
26 kDa KRAS
43 kDa β-actin
g
H1299 (NRAS^{Q61K}) xenografts
VHL-DP KRAS
No dox Dox
55 kDa pAKT^{S473}
55 kDa AKT
43 kDa pMEK1/2
43 kDa MEK1/2
43 kDa pERK1/2
43 kDa ERK1/2
43 kDa FLAG (DP)
26 kDa pan-RAS
26 kDa KRAS
43 kDa β-actin

Supplementary Figure 9: Characterisation of H358-FLuc and H1299-FLuc clones and effect of the degraders on H1299 tumours xenografts. (a) Western blot analysis of the RAS downstream signalling pathways RAS/RAF/MEK/ERK and PI3K/AKT in H1299-FLuc and H358-FLuc cloned cells. FLAG antibody is used to show expression of iDAbs and DARPins when induced with 0.2 $\mu g.mL^{-1}$ of doxycycline for 72 hours ((+) or not induced (-)). KRAS, NRAS and HRAS protein levels were also assessed by Western blot to confirm the proteolysis of the degraders target(s). β-actin is the loading control. The black arrowheads indicate the specific band corresponding to $pAKT^{S473}$ and NRAS proteins. (b, c) Cell growth assay of each clone for H358 (b) and H1299 (c). Cells were grown with or without 0.2 $\mu g.mL^{-1}$ of dox and counted every two days for 6 days to determine their growth. Each experiment (a, b, c) was performed twice. Error bars in (b, c) are mean ± SD from two independent biological repeats. (d, e) $5x10^6$ H1299 cells inducibly expressing either FLuc/iDAb RAS-UBOX (d) or FLuc/VHL-DP KRAS (e) were injected subcutaneously into CD-1 nude mice. After tumours reached 2-3 mm diameter, animals were separated into groups of 3-5 mice and treated or not with doxycycline (+/- dox) in drink and food. Tumour burden was assessed by bioluminescence imaging at the end of the experiment (day 20). Photon flux (i.e. luminescence signal) was quantified for each group at day 20 (mean ± SEM) from n=5 mice (d) and n=2 or 3 mice for the no dox and dox conditions respectively (e). The colour scale used in d and e is the same as in c. Statistical analyses were performed with an unpaired two-tail Student's t test: *P = 0.0264 and ns: non-significant. (f, g) Western blot analysis of H1299 tumour lysates from xenograft resected from mice treated 20 days with -/+ doxycycline in H1299-FLuc/iDAb RAS-UBOX (f) and H1299-FLuc/VHL-DP KRAS (g) mice. Each experiment (f, g) was performed once. The black arrowhead indicates the specific band corresponding to FLAG-tagged VHL-DP KRAS protein. **a-g** Source data are provided as a Source Data file.
