# Supplementary material for: A potent KRAS macromolecule degrader specifically targeting tumours with mutant KRAS
Source: Nat Commun. 2020 Jun 26;11:3233. doi: 10.1038/s41467-020-17022-w (PMC7319959; doi:10.1038/s41467-020-17022-w)
Supplement: Supplementary file 3 — Reporting Summary [file 41467_2020_17022_MOESM3_ESM.pdf]

nature research

Corresponding author(s): Terry Rabbitts

Last updated by author(s): May 21, 2020

Reporting Summary

Nature Research wishes to improve the reproducibility of the work that we publish. This form provides structure for consistency and transparency in reporting. For further information on Nature Research policies, see Authors & Referees and the Editorial Policy Checklist .

Statistics

For all statistical analyses, confirm that the following items are present in the figure legend, table legend, main text, or Methods section.

n/a	Confirmed	
☐	☒	The exact sample size (*n*) for each experimental group/condition, given as a discrete number and unit of measurement
☐	☒	A statement on whether measurements were taken from distinct samples or whether the same sample was measured repeatedly
☐	☒	The statistical test(s) used AND whether they are one- or two-sided<br>*Only common tests should be described solely by name; describe more complex techniques in the Methods section.*
☒	☐	A description of all covariates tested
☒	☐	A description of any assumptions or corrections, such as tests of normality and adjustment for multiple comparisons
☐	☒	A full description of the statistical parameters including central tendency (e.g. means) or other basic estimates (e.g. regression coefficient) AND variation (e.g. standard deviation) or associated estimates of uncertainty (e.g. confidence intervals)
☐	☒	For null hypothesis testing, the test statistic (e.g. *F*, *t*, *r*) with confidence intervals, effect sizes, degrees of freedom and *P* value noted<br>*Give P values as exact values whenever suitable.*
☒	☐	For Bayesian analysis, information on the choice of priors and Markov chain Monte Carlo settings
☒	☐	For hierarchical and complex designs, identification of the appropriate level for tests and full reporting of outcomes
☒	☐	Estimates of effect sizes (e.g. Cohen's *d*, Pearson's *r*), indicating how they were calculated

Our web collection on statistics for biologists contains articles on many of the points above.

Software and code

Policy information about availability of computer code

Data collection	No software was used
Data analysis	Data quantifications and analyses were performed using Image Lab (Biorad), Prism 8.0 (GraphPad Software) or Living Image (PerkinElmer).

For manuscripts utilizing custom algorithms or software that are central to the research but not yet described in published literature, software must be made available to editors/reviewers. We strongly encourage code deposition in a community repository (e.g. GitHub). See the Nature Research guidelines for submitting code & software for further information.

Data

Policy information about availability of data

All manuscripts must include a data availability statement. This statement should provide the following information, where applicable:

- Accession codes, unique identifiers, or web links for publicly available datasets
- A list of figures that have associated raw data
- A description of any restrictions on data availability

All relevant data are within the paper and its Supplementary Information file and in a Source data file. The source data underlying Figures 1a-f, 2, 3, 4, 5, 6, 7, 8 and Supplementary Figures 1b-d, 5, 6, 7, 8 and 9 are provided as a Source Data file. Additional data supporting the conclusions are available from the corresponding author on reasonable request.

Field-specific reporting

Please select the one below that is the best fit for your research. If you are not sure, read the appropriate sections before making your selection.

Life sciences study design

All studies must disclose on these points even when the disclosure is negative.

Sample size	This is only relevant to the mouse studies. No sample size calculations were performed. The number of mice used was based on our experience with the models. The sample sizes were sufficient as we reached statistical significance.
Data exclusions	One mouse of each of the no doxycycline control groups injected with H358-FLuc/VHL-DP KRAS, H358-FLuc/iDAb RAS-UBOX and H1299-FLuc/VHL-DP KRAS was excluded from analysis due to lack of tumour development.
Replication	Reproducibility was verified by repeating the experiments. The number of repeats are stated in the figure legends.
Randomization	This is only relevant to the mouse experiments. Yes allocation was random.
Blinding	No blinding was used as the experimenter who planned the experiment also performed the experiment.

Reporting for specific materials, systems and methods

We require information from authors about some types of materials, experimental systems and methods used in many studies. Here, indicate whether each material, system or method listed is relevant to your study. If you are not sure if a list item applies to your research, read the appropriate section before selecting a response.

Materials & experimental systems

n/a	Involved in the study	
☐	☒	Antibodies
☐	☒	Eukaryotic cell lines
☒	☐	Palaeontology
☐	☒	Animals and other organisms
☒	☐	Human research participants
☒	☐	Clinical data

Methods

n/a	Involved in the study	
☒	☐	ChIP-seq
☒	☐	Flow cytometry
☒	☐	MRI-based neuroimaging

Antibodies

Antibodies used

Phospho-ERK 1/2, Rabbit antibody, Cell Signaling Technology, Cat#9101S RRID:AB_331646
Total ERK 1/2, Rabbit antibody, Cell Signaling Technology, Cat#9102S RRID:AB_330744
Phospho-MEK 1/2, Rabbit antibody, Cell Signaling Technology, Cat#9154S RRID:AB_2138017
Total MEK 1/2, Mouse antibody, Cell Signaling Technology, Cat#4694S RRID:AB_10695868
Phospho-AKT S473, Rabbit antibody, Cell Signaling Technology, Cat#4058S RRID:AB_331168
Total AKT, Rabbit antibody, Cell Signaling Technology, Cat#9272S RRID:AB_329827
GFP, Mouse antibody, Santa Cruz Biotechnology, Cat#sc-9996 RRID:AB_627695
β-Actin, Mouse antibody, Sigma-Aldrich, Cat#A1978 RRID:AB_476692
Pan-RAS, Mouse antibody, Millipore, Cat#OP40 RRID:AB_213400
KRAS, Mouse antibody, Santa Cruz Biotechnology, Cat#sc-30 RRID:AB_627865
NRAS, Mouse antibody, Santa Cruz Biotechnology, Cat#sc-31 RRID:AB_628041 now discontinued so then use of NRAS, Goat antibody, Abcam, Cat#ab77392 RRID:AB_1524048
HRAS, Rabbit antibody, Proteintech, Cat#18295-1-AP RRID:AB_2121046
Cleaved PARP, Rabbit antibody, Cell Signaling Technology, Cat#9541 RRID:AB_331426
Cleaved Caspase 3, Rabbit antibody, Cell Signaling Technology, Cat#9664 RRID:AB_2070042
α−tubulin, Rabbit antibody, Abcam, Cat#ab4074 RRID:AB_228800
FLAG tag, Mouse antibody, Sigma, Cat#F3165 RRID:AB_259529

Validation

All these antibodies have been validated for western blot use (see manufacturers website).

Eukaryotic cell lines

Policy information about cell lines

Cell line source(s)

HEK293T, ATCC, Cat#CRL-3216 RRID:CVCL_0063
HCT116, ATCC, Cat# CCL-247, RRID:CVCL_0291
HT1080, ATCC, Cat# CCL-121, RRID:CVCL_0317
H358, ATCC, Cat# CRL-5807, RRID:CVCL_1559
A549, ATCC, Cat# CCL-185, RRID:CVCL_0023

	HCC827, ATCC, Cat# CRL-2868, RRID:CVCL_2063<br>MRC5, T24 and H1299 were a gift from Prof. Geoff Higgins, University of Oxford and MIA PaCa2 from Prof Gillies McKenna.
Authentication	Mutation detection of RAS mutations using RT-PCR for the cancer cell lines and MIA PaCa2 was authenticated by short-tandem repeat (STR) DNA profiling services (ATTC)
Mycoplasma contamination	All cell lines tested negative for mycoplasma contamination.
Commonly misidentified lines<br>(See ICLAC register)	No commonly misidentified cell lines were used in the study.

Animals and other organisms

Policy information about studies involving animals; ARRIVE guidelines recommended for reporting animal research

Laboratory animals	For all xenograft experiments, 5-7-week-old female CD-1 athymic nude mice (Charles River) were used.
Wild animals	The study did not involve wild animals.
Field-collected samples	The study did not involve field-collected samples.
Ethics oversight	The University of Oxford Ethical Review Committee approved the study protocol described in the manuscript.

Note that full information on the approval of the study protocol must also be provided in the manuscript.
